# A Critical Appraisal of the Most Recent Investigations on Ora-Pro-Nobis (*Pereskia* sp.): Economical, Botanical, Phytochemical, Nutritional, and Ethnopharmacological Aspects

**DOI:** 10.3390/plants12223874

**Published:** 2023-11-16

**Authors:** Valéria Maria Costa Teixeira, Anielle de Oliveira, Emanueli Backes, Cristina Giatti Marques de Souza, Rafael Castoldi, Anacharis Babeto de Sá-Nakanishi, Lívia Bracht, Jurandir Fernando Comar, Rúbia Carvalho Gomes Corrêa, Fernanda Vitória Leimann, Adelar Bracht, Rosane Marina Peralta

**Affiliations:** 1 Department of Biochemistry, State University of Maringá, Maringá 87020-900, Brazil; valeriamariateixeira@gmail.com (V.M.C.T.); ani.028@hotmail.com (A.d.O.); emanuelibackes@outlook.com (E.B.); cgmsouza@uem.br (C.G.M.d.S.); rcastoldi@uem.br (R.C.); absnakanishi@uem.br (A.B.d.S.-N.); lbracht@uem.br (L.B.); jfcomar@uem.br (J.F.C.); abracht@uem.br (A.B.); 2 Post-Graduate Program in Clean Technologies, Cesumar Institute for Science, Technology and Innovation—ICETI, Cesumar University—UNICESUMAR, Maringá 87050-900, Brazil; rubia.correa@unicesumar.edu.br; 3 Food Departament, Federal University of Technology-PR, Campus of Campo Mourão, Campo Mourão 87301-899, Brazil; fernandaleimann@utfpr.edu.br

**Keywords:** bioactive properties, extract, mucilage, polysaccharide, protein, terpenoids, polyphenols, foods, extraction methods, essential oils

## Abstract

*Pereskia aculeata* Miller and *Pereskia grandfolia* Haw, known as ‘ora-pro-nobis’, are unconventional vegetables belonging to the Cactaceae family, native to the Americas and common in the northeast and southeast regions of Brazil. This review attempts to present a balanced account of both the methods used for obtaining extracts from the diverse parts of the plants and the results that were obtained in terms of their applicability to foods and other products with biological activities. Attention will also be devoted to the properties of their bioactives and their applications to real food products. Methods for obtaining extracts from the diverse parts of the plants will be analyzed, as well as the chemical nature of the bioactives that were hitherto identified. Next, the applicability of ora-pro-nobis in either its integral form or in the form of extracts or other products (mucilages) to the production of food and dietary supplements will be analyzed. The species have been extensively investigated during the last few decades. But, the determination of chemical structures is frequently incomplete and there is a need for new studies on texture determination and color evaluation. Further studies exploring the fruit and flowers of *P. aculeata* are also required.

## 1. Introduction

The two edible species of the genus *Pereskia*, *P. grandifolia* Haworth and *P. aculeata* Miller, popularly known as ora-pro-nobis, belong to the Cactaceae family and Pereskioideae subfamily. They are native to South America and adaptable only to low altitudes [1,2,3,4].

In Brazil, as well as in several South American countries, ora-pro-nobis leaves are important both as food and as folk medicines. Ora-pro-nobis leaves are used as ingredients in various sweet and savory dishes. Especially in low-income communities, they are known as the “meat of the poor” because they are frequently the main affordable protein source [5,6]. It has been reported that in the city of Diamantina, Minas Gerais State, almost 80% of the population eats this nutritious protein-rich vegetable on a regular basis. *P. aculeata* leaves can be used in the preparation of salads, soups, omelets, and pies, and the leaf flour can serve as an enriching element in the formulation of breads, cakes, and pastas [2]. Furthermore, its mucilage can substitute eggs in food preparations [7], which may be especially convenient for consumers with food allergies or diet restrictions. *P. aculeata* fruits can be applied in the manufacture of juices, jellies, mousses, and liquors, and their seeds can be germinated to produce edible shoots [2].

The benefits of ora-pro-nobis in the folk medicine realm have been appreciated for centuries. Traditionally, ora-pro-nobis preparations are believed to be effective in the treatment of renal disorders (with no signs of toxicity), capable of healing skin wounds and mildening inflammatory processes, and able to act as an effective emollient [1]. There are also reports of the utilization of certain species of the genus *Pereskia* as a natural medicine against cancer [8], which are to some degree corroborated by studies showing antiproliferative activity against several malignant cells [9]. Other studies attribute in vivo topical anti-inflammatory [10], antinociceptive [11], and antioxidant activities [8,12,13,14] as well as antimicrobial potentials [9] to ora-pro-nobis.

Taking into account the great potential of “ora-pro-nobis” in the food and natural medicine sectors, as briefly outlined above, it seems that a detailed account of all these properties could be of great interest to the scientific community. This review, therefore, aims at describing and analyzing the many biological and chemical properties of *P. aculeata* and *P. grandifolia*. Special attention will be devoted to the properties of their bioactives and their applications to real food products. Unlike the most recent reviews published on ora-pro-nobis [4,15], the present work attempts to present a balanced account of both the methods used for obtaining extracts from the diverse parts of the plants (leaf, fruit, seed, etc.) and the results that were obtained in terms of their applicability to foods and other products with biological activities. Chemical structures will be analyzed mainly in the context of their possible biological effects and relevance for methodological approaches, as a more exhaustive structural description can be found in a previous work [16]. Finally, the applicability of ora-pro-nobis in either its integral form or in the form of extracts or other products (mucilages) to the production of food and dietary supplements will be analyzed. We hope that this work will result in a source of valuable information about the aforementioned ora-pro-nobis species.

## 2. Search Methodology

**Figure 1:**
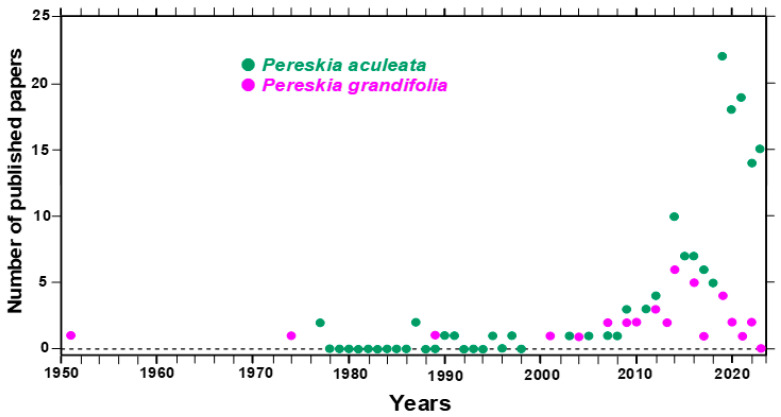
Number of published papers found under the keywords “*Pereskia* AND *aculeata*” or “*Pereskia* AND *grandifolia*” by year since 1950 (Scopus).

Initially, the literature (articles) from 1950 to 2023 was searched in the Scopus Database under the keywords “*Pereskia* AND *aculeata*” and “*Pereskia* AND *grandifolia*”. This search was restricted to title, abstract, and keywords. As a second step, the literature search was carried out in the Google Scholar Database under the following keywords: *Pereskia*; *aculeata; grandifolia;* extract; bioactive; food; flour; mucilage; rheology; phenolic compounds; vitamin; carotenoids; mineral; leaves; flower; fruit. This search also includes Master’s and Ph.D. theses, most of them from Brazil. Figure 1 offers an overview of the evolution of the recorded studies when the keywords “*Pereskia* AND *aculeata*” or “*Pereskia* AND *grandifolia*” were searched in the Scopus Database. The first study on ora-pro-nobis, which was done with *Pereskia grandifolia*, was published in 1951 [17]. It consists of a report about the cardiovascular effects of the plant. The first two studies about the other ora-pro-nobis species, *Pereskia aculeata*, were published in 1997 [18,19], and they were concerned with protein extraction and nutritional value. After these pioneering studies, relatively few investigations have explored the ora-pro-nobis potential up until the last decade. After the year 2009, however, there was a significant increase in studies in which the ora-pro-nobis plants were analyzed from several angles and viewpoints including their nutritional potential [20], fruit carotenoids profile and total polyphenols [3], mucilage extraction [21], bioactivity and chemical characterization [13,14], and the use of the leaves to enrich foods [22]. All these and other aspects that were approached by several additional studies will here be detailed and analyzed in separate sections and subsections.

## 3. Morphology, Chemical Characteristics, and Cultivation

**Figure 2:**
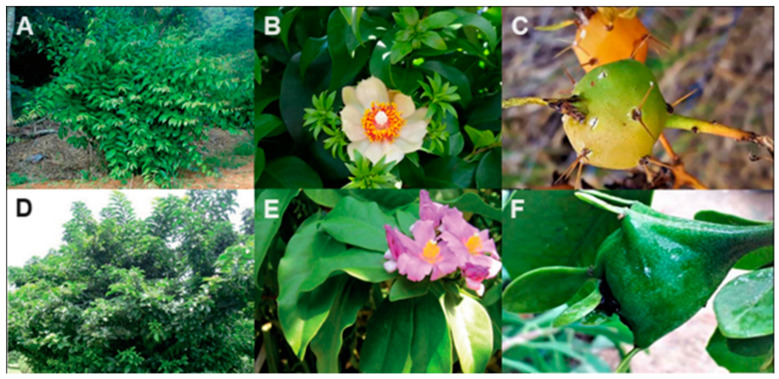
*Pereskia aculeata* Miller (**A**); flower and leaves; (**B**) fruit (**C**); *Pereskia grandifolia* Haw (**D**); flower and leaves (**E**); fruit (**F**).

The genus *Pereskia* includes 17 species that occur in arid or semi-arid regions. The species are widely distributed in the Caribbean and Central and South America, all varieties of dry forest habitats [23]. In Brazil, the two species, *Pereskia aculeata* and *Pereskia grandifolia,* are native to the Atlantic Forest (Figure 2). The flowers of the first species are yellow, whereas the second has purple ones [2].

The genus *Pereskia* is included in the Cactaceae family, and comprises 17 species: *P. aculeata*, *P. aureiflora* F. Ritter, *P. bahiensis* Gürke, *P. bleo* (Kunth) DC., *P. diazromeroana* Cárdenas, *P. guamacho*, *P. grandifolia* Haw., *P. horrid* DC., *P. lychnidiflora* DC., *P. marquenoi* Areces, *P. nemorosa* Rojas Acosta, *P. portulacifolia* (L.) DC., *P. quisqueyana* Alain, *P. sacharosa* Griseb, *P. stenantha* F. Ritter, *P. weberiana* K. Schum, and *P. zinniiflora* DC. [1,24]. These species behave like climbers or shrubs. They have a thin stem and long branches with distributed needles; elliptical, flat, and fleshy leaves; and abundant flowers. The petals display varying shades according to the species [25]. The generally succulent leaves are indicative of the presence of mucilage, as indeed confirmed by chemical studies, as will be outlined in one of the following sections. Solitary terminal flowers or short hermaphrodite crests may appear at the end of the branches [26,27]. After flowering, they produce fruits in small, rounded berries.

*Pereskia* flowers are of the perigynous type and have a hypanthium with bracteoles and needles. Flowering, although abundant, is ephemeral and lasts only one day, attracting many pollinators, mainly bees. The fruit is pomaceous, with a juicy hypanthium, pericarp, and seeds immersed in a yellowish gelatinous mass. The seed is exotestal and develops from an amphitropous, bythegmic, and crassinucellate ovule [25,28]. The young fruit consists of hypanthium, pericarp, and seeds [28]. The hypanthium remains green and has bracteoles, also green, and areoles, where there are spikes and hairs. After ripening, the fruit presents a yellow-orange hypanthium and may completely lose its bracteoles and needles.

The fruits of *P. aculeata* are small, spherical yellow thorny berries, yellow or orange in color when ripe (Figure 2C) [29,30,31]. The fruits of *P. grandifolia* have a pyriform berry shape, flat or angular and with bracts. When immature, they do not have funicular pulp (Figure 2F) [12].

An important feature of both *P. aculeata* and *P. grandifolia* is that they are fairly drought-resistant [29]. *P. aculeata* is a perennial and shrubby species (Figure 2A) that grows in tropical, subtropical, and hot temperate regions. It prefers dry and semi-arid habitats but can also thrive in moist to sub-humid evergreen forests. It adapts to a wide range of soil types, including fine sand, sandy loam, limestone and granite soils, and rocky areas with a pH in the range of 6 to 7.5 [32,33]. *P. grandifolia* (Figure 2D) is a species native to the Atlantic Forest, and it occurs from the northeast states to southern Brazil. It is shrubby and can reach between 3 and 6 m in height. It has well-developed woody stems and thorns, along with its fleshy (succulent) overlapping leaves and terminal flowers arranged in a ridge [34].

The approximate compositions of the *P. aculeata* and *P. grandifolia* leaves and fruits were determined in several studies and are presented in Table 1 and Table 2. The data in reference [35], which refer to *P. aculeata,* are actually an average of results compiled from several sources. Fresh leaves show remarkable vitamin levels (vitamin A, vitamin C, and folic acid) and high levels of total dietary fiber, as well as considerable amounts of minerals (calcium, magnesium, manganese, and zinc), revealing the nutritional benefits of ora-pro-nobis leaves. The fruits of *P. aculeata* were characterized with respect to titratable acidity, total soluble solids, and vitamin C content [12], the results being equal to 1.17 ± 0.3 (% citric acid), 3.2 ± 0.7 (Brix) and 12.06 ± 1.30 (mg/100 g), respectively. No data on the composition of *P. grandifolia* fruits was found in the searched literature.

**Table 1:** Approximate composition of ora-pro-nobis species.

Ora-Pro-Nobis Species and Samples	Moisture (%)	Total Proteins (%)	Lipids (%)	Ash (%)	Carbohydrates (%)	Total Dietary Fiber (%)	Reference
*P. aculeata* fresh leaves	89.5 ± 0.2	28.4 ± 0.4	4.1 ± 0.3	16.1 ± 0.1	nd.	39.1	[20]
*P. aculeata* (dry basis) leaves *	8.3	23	4	16.6	43.7	30.2	[35]
*P. aculeata* (dry basis) leaves	12.46 ± 0.47	28.99 ± 0.59	5.07 ± 0.15	14.81 ± 0.18	29.53 ± 1.28	21.60 ± 0.82	[26]
*P. grandifolia* (dry basis) leaves	10.94 ± 0.78	32.02 ± 0.46	6.72 ± 0.30	18.8 ±0.92	29.86 ± 1.32	18.82 ± 0.92	[26]
*P. grandifolia* fresh leaves	86.50 ± 0.13	14.64 ± 0.87	2.97 ± 0.86	nd.	24.47 ± 0.35	nd.	[36]
*P. aculeata* fruit	87.37 ± 0.26	---	0.23 ± 0.05	0.93 ± 0.01	11.47 ± 0.22	nd.	[37]

* Average results from several studies; nd, not determined.

The protein content and the amino acid profile of five ora-pro-nobis clones (*P. aculeata*), selected from the Brazilian Agricultural Research Corporation (*Embrapa*) vegetables collection, were evaluated [38]. It was concluded that ora-pro-nobis constitutes a good source of protein, with a relevant amino acids profile from the nutritional point of view, especially with reference to leucine, phenylalanine, and lysine.

**Table 2:** Mineral and vitamin contents of ora-pro-nobis.

Ora-Pro-Nobis Species and Samples	Mineral Content (mg/100 g)	Vitamin Content (mg/100 g)	Reference
*P. aculeata* (fresh leaves)	Calcium= 3420 Magnesium = 1900 Potassium = 1632 Phosphorus = 156 Manganese = 4.2 Boron = 5.5 Copper = 1.4	β-carotene = 4.2 ± 0.2 Vitamin A = 2333 IU/100 g Vitamin C = 185.8 ± 14.0 Folic acid = 19.3	[20]
*P. aculeata* (dry basis, leaves) *	Iron = 19.8 Zinc = 5.7 Calcium = 2679.3 Magnesium = 1065.3 Phosphorous 447.4 Potassium = 3266 Copper = 0.7 Boron = 4.1 Manganese = 23.6 Sulphur = 640.8	Total carotenoids = 186 µg/g	[35]
*P. aculeata* (dry basis, leaves)	n.a.	Thiamine (B1) = 1.18 ± 0.37 Nicotinamide (B3) = 2.75 ± 0.39 Pantothenic acid (B5) = 0.47 ± 0.43 Biotin (B7) = 35.12 ± 7.26 Ascorbic acid = 0.83 ± 0.14	[39]

* Average results from several studies; n.a., not analyzed.

Although ora-pro-nobis plays an important role in South African folk medicine, in this country, it has been considered an invasive alien plant (*P. aculeata*, specifically) [40,41]. The plant was first recorded in the Cape Town botanical gardens in 1858. It has since spread across South Africa, dominating previously pristine, indigenous vegetation, and driving biodiversity loss. Since *P. aculeata* can become intertwined with native vegetation, chemical and mechanical controls are ineffective, and biological control is considered the sole option [42]. Two species have been released to date in South Africa for *P. aculeata* biological control, namely the leaf-chewing beetle *Phenrica guerini* Bechyné (Chrysomelidae: Coleoptera) in 1991, and the stem-wilting bug *Catorhintha schaffneri* Brailovsky & Garcia (Coreidae: Hemiptera) in 2014 [43]. Evaluation of the growth of *P. aculeata* under different CO_2_ concentrations and watering regimes allowed researchers to conclude that increasing atmospheric CO_2_ appears unlikely to have been a major contributor to the invasion success of *P. aculeata* in South Africa [43].

In relation to ora-pro-nobis cultivation, it is worth commenting on two germination studies. *P. aculeata* seeds were germinated, and the plants were cultivated under artificial shade, half shade, and without shade [44]. Growth was evaluated in terms of mass and structural units and the mineral residue, proteins, lipids, neutral detergent fiber, phenols, and antioxidant activity were quantified. It was found that the plants that were submitted to total shade showed higher ash, lipid, and protein content and that for all evaluated parameters, the quantified levels in leaves were higher than in stems. The optimal temperature for *P. aculeata* and *P. grandifolia* seed germination was determined under various germination factors [45]. The best germination response of the *P. aculeata* and *P. grandifolia* seeds was observed at 30 °C and 33 °C, respectively. The germination performance as a function of temperature can be described by a Weibull distribution model with three parameters.

## 4. Folk Medicinal Claims

In folk medicine, *Pereskia* leaves have been used for various purposes depending on the geographic location because genetical, environmental, and physiological conditions can influence composition and, consequently, biological activities [3]. In Malaysian traditional medicine, the *P. grandifolia* leaves are employed in the treatment of cancer, diabetes, hypertension, and diseases associated with inflammation and rheumatism, as well as in the relief of gastric pain, treatment of ulcers, and body rejuvenation [46,47]. In Brazil, the leaves are integrated into folk medicine as emollients for treating skin rashes, and the fruits have been claimed to possess expectorant and antisyphilitic properties [1]. The acute toxicity of *P. aculeata* has been considered neglectable based on data obtained in a rat model and on the cytogenotoxicity lettuce model [48]. These observations, in principle, disprove toxicity as an argument against the use of the plant as a functional food.

Based on the metabolic profile and intestinal motility in Wistar rats fed with *P. aculeata* flour, it was concluded that the latter may bring considerable health benefits [49]. The improved intestinal motility was associated with reduced visceral fat and an improved lipid profile, including increased HDL-c levels. The opinion was expressed that the incorporation of this flour into industrial products may represent a convenient and effective strategy for stimulating the intake of healthier products [49].

In several instances, ora-pro-nobis has been considered a superfood [6] and an Unconventional Food Plant (UFP), food classes recognized as important complementary sources for fighting nutritional deficiencies [50]. *P. aculeata* leaves can be used in the preparation of salads, soups, and pies. Leaf flour can serve as a complementary or ideal element for breads, cakes, and dough. The fruits of *P. aculeata* can be used for making juices, jellies, mousses, and liqueurs. Justifying all these uses is the fact that the leaves are important sources of protein and also contain minerals, fibers, and vitamins A and C, in addition to folic acid [9], microelements, β-carotene, and ascorbic acid [20], as illustrated by Table 1 and Table 2. The consumption of ora-pro-nobis leaves has also been associated with the prevention of iron deficiency, anemia, osteoporosis, cancer, and constipation [7].

## 5. Development of Food Products Added with Ora-Pro-Nobis Flour

The flour of ora-pro-nobis leaves has been used in various food product formulations, as displayed in Table 3. Most of the studies listed in Table 3 evaluated only the approximate composition and sensory acceptance of the food formulated with ora-pro-nobis flour. The work that describes the development of a milk-based beverage containing ora-pro-nobis flour, however, presents a more in-depth study of the health benefits associated with its consumption [22]. These authors evaluated the effect of the beverage on the intestinal microbiota, gastrointestinal symptoms, and anthropometric parameters in women. The beverage reduced the weight, waist circumference, and body fat of the participants. In addition, the treatment increased satiety, reduced eructation and constipation, and improved feces consistency.

A Brazilian peanut candy (locally known as *paçoca*) was prepared using ora-pro-nobis flour [51]. For comparative purposes, similar peanut-based and cashew-nut-based control formulations of *paçoca* were prepared. The sensory evaluation revealed an acceptability in the range of 74% to 75% for the formulations with added ora-pro-nobis flour. An extruded corn snack with the addition of ora-pro-nobis flour (*P. grandifolia*) was prepared with the purpose of increasing its biological value in terms of proteins and fibers [52]. Two formulations were made with the addition of 10 and 20% leaf flour, as well as a control sample composed of 100% corn grits. The best sensory acceptability was found for the formulation containing 10% ora-pro-nobis flour. Increasing the protein content was also the purpose of the addition of ora-pro-nobis flour to two different cereal bar formulations in which pineapple peel was used to produce the binder syrup [53]. The addition of the flour doubled the protein levels when compared to the traditional cereal bars.

**Table 3:** Food products enriched with ora-pro-nobis leaf flour.

Food Product	Product Characterization	Reference
Yogurt	Approximate composition, pH, *Lactobacillus acidophilus* and *Bifidobacterium* BB-12 counting, and sensory analysis (acceptance test)	[54]
Milk-based beverage	Approximate composition, total phenolics, antioxidant activity, clinical trial (analysis of fecal microbiota, gastrointestinal symptoms, and anthropometric parameters)	[22]
Talharim pasta	Sensory analysis (acceptance and preference tests) and proximate composition	[55]
Bread	Approximate composition and sensory analysis (acceptance test)	[56]
Bread	Sensory acceptance test and purchase intention	
Cake	Protein content and sensory evaluation (acceptance test)	[57]
Bread and pie	Sensory acceptance test	[58]
Bread	Sensory acceptance test	[59]
Veggie pie	Sensory acceptance test	[60]
Cake	Approximate composition	[61]
Hot dog	Approximate composition, color, pH, texture profile analysis, and sensory acceptance tests	[62]
Extruded corn snack	Approximate composition and sensory analysis (acceptance test)	[52]
Vegan chickpea burger	Approximate composition, color, and sensory analysis (acceptance test)	[63]
Cereal bar	Protein content and sensory evaluation (acceptance test)	[53]
*Paçoca* (peanut candy)	Sensory acceptance test	[51]
Rice dumplings (leaves in natura)	Approximate composition and sensory analysis (acceptance test)	[64]

Rice dumplings were prepared with the addition of ora-pro-nobis leaves in natura (0%—standard, 2.5%, and 5%) [64]. There were high levels of similar acceptability among the dumpling formulations in the sensory evaluation. As expected, the formulations containing ora-pro-nobis had higher protein content, but also lower water content when compared to the control dumplings. In contrast, in cakes to which in natura ora-pro-nobis leaves were added, increased water retention was observed in parallel with increased crude fiber and total calorie content [61].

An evaluation of the sensory acceptance and purchase intention of a veggie pie to which ora-pro-nobis was added (10%) was done [60]. The comparison basis was provided by two standards, namely pies prepared with traditional wheat flour and whole-wheat flour. In general, the three cake formulations were well-accepted by consumers. The sample with added ora-pro-nobis flour was well-accepted in terms of flavor, but it was not well-accepted in relation to its appearance due to its green color. Still within the sector of acceptability, four different vegan chickpea burger formulations were developed with the addition of ora-pro-nobis [63]. The best-evaluated formulations were those with the addition of 10% ora-pro-nobis and the control. It was noted that the incorporation of ora-pro-nobis leaves resulted in increases in moisture and water activity, without altering the acidity (pH).

The behavior of ora-pro-nobis leaf flour with high fiber and protein content when added to sausage formulations was analyzed [62]. Contents of 1% and 2% were tested. The sausages underwent changes in color and texture; however, the flour did not affect the sensory acceptance. It was concluded that ora-pro-nobis leaf flour can be used at a percentage of up to 2% in sausage formulations in order to increase their nutritional quality.

A critical examination of the studies listed in Table 3 reveals that sensory aspects, as well as the approximate composition, were the main priorities of the authors. Other characteristics and properties received much less attention. The texture of the products, for example, was evaluated in a single study [62]. Although sensory acceptability involves texture concepts, the results are subjective, while texture profile analysis results are objective [65]. Approaches to bioactive properties, digestibility, shelf life, etc., are lacking in the studies listed in Table 3.

One of the main problems in exploring vegetable leaves as a source of nutrients is the presence of anti-nutritional factors, such as trypsin inhibitors, oxalates, and tannins. These compounds may be toxic and/or interfere with the digestibility and bioavailability of some nutrients, such as proteins and minerals. In this respect, it is worth mentioning the study in which the amino acid profile of *P. aculeata* and *P. grandifolia* leaves was evaluated in addition to the effects of heating media and time on several parameters [66]. These parameters included, among others, the total content of dietary fiber, minerals, oxalic acid, and tannins, and the possibility of trypsin inhibition. It was found that thermal treatments decreased the total dietary fiber and mineral levels in both species. Furthermore, wet cooking was more effective in maintaining dietary fiber and mineral levels. The conclusion was that there is great anti-nutritional retention in routine situations of preparation and consumption.

## 6. Extraction of the Bioactives of *P. aculeata* and *P. grandifolia*

Ora-pro-nobis extracts have been prepared using various techniques and solvents, as detailed in Table 4. The best methods and procedures are those that combine strategies for the maximal recovery of the desired active principles, thus aggregating value to the plant material without extracting or generating anti-nutritional factors. One of the main purposes of these extraction processes was to obtain mucilage materials to be used as emulsifiers and polymeric materials for films. A further purpose was the isolation of bioactive compounds, basically of low molecular weight, that can be used as bio-preservatives and for food fortification purposes.

As can be deduced from the information presented in Table 4, most of the extraction studies involved the leaves of the *P. aculeata* species. Fruit extraction was done in just a few studies and flower extraction seems not to have been done up to now. This indicates that there is still a lot of information to be gained from ora-pro-nobis. Bioactive extracts were frequently characterized in terms of their antioxidant activity. Additionally, cytotoxic effects on cancer and normal cell lines [8,9,49,67,68], anti-inflammatory activity [11], and cholinergic enzyme inhibitory capacity [36] have also been investigated. Aqueous and hydroalcoholic extracts of *P. grandifolia* leaves had their antibacterial activity tested against *S. aureus* and *P. aeruginosa* [69], and the authors found that extracts showed inhibitory activity against the tested bacteria. However, only the hydroalcoholic extract obtained by reflux was bactericidal at the tested concentrations. Concerning mucilage extraction, most of the studies applied the hot homogenization technique using water as a solvent.

Encapsulation of an ora-pro-nobis extract was performed just in a single study [68], in which a self-assembling electrostatic method was applied. The encapsulating agents were pectin and chitosan. Based on cytotoxicity (Caco-2 cell) experiments, the authors stated that the encapsulated extract presented a cell viability above ~80% and cell iron uptake at levels slightly close to particles prepared using FeSO_4_, confirming a good absorption property in vitro. In another work, an ora-pro-nobis extract as the encapsulating agent for α-tocopherol, together with whey protein isolated by free-drying, was used [70]. The α-tocopherol bioaccessibility evaluation was carried out by mimetizing the human physiological conditions. Analysis by HPLC indicated that the encapsulating material presented good protection for α-tocopherol against the environment. These few studies indicate that the encapsulation of ora-pro-nobis extracts is certainly a promising topic, but much more in-depth and detailed studies are needed to arrive at definitive conclusions about applicability.

**Table 4:** Extraction methods and bioactive/functional characterization of *P. aculeata* and *P. grandifolia* extracts.

*Pereskia* sp. Plant Part	Extraction Technique	Extraction Solvent	Bioactive/Functional Characterization	Reference
*P. aculeata* leaves	Hot extraction, pressing, and precipitation	Water	Mucilage: emulsifier	[21]
*P. aculeata* green fruit	Pressing and precipitation	None	Mucilage: emulsifier	[71]
*P. aculeata* fruits (unripe, intermediate, and ripe)	Homogenization with water	Water	Fruit pulp: antioxidant activity (DPPH, ABTS, ORAC, and total phenolic compounds), total carotenoids, total chlorophylls	[72]
*P. aculeata* leaves	Simple stirring	Water, ethanol, and acetone	Bioactive extract: in vitro antioxidant and antihemolytic activities	[73]
*P. aculeata* leaves	Sequential solvent extraction and hydro-distillation	Petroleum ether followed by chloroform and methanolWater	Bioactive extracts and essential oil: antimicrobial (*Staphylococcus aureus*, *Bacillus cereus*, and *Bacillus cereus*, *Escherichia coli*, *Pseudomonas aeru-ginosa*, *Penicillium citrinum*, *P. ex-pansum and Aspergillus versicolor*), antioxidant, cytotoxicity on neuro-blastoma SH-SY5Y cancer cell line and influence on adenylate cyclase (ADCY) expression	[9,45]
*P. aculeata* leaves	Simple stirring	Ethanol:water (70:30)	Bioactive extract: antioxidant (OxHLIA, TBARS, DPPH, and ABTS) and hepatotoxicity	[6]
*P. aculeata* leaves	Homogenization and hot extraction or pressing	Water	Mucilage: emulsifier	[74]
*P. aculeata* leaves	Hot extraction, pressing, and precipitation	Water	Mucilage: emulsifier applied to Petit Suisse cheeses	[75]
*P. aculeata* leaves	Turbo-extraction	Ethanol:water (95:5)	Bioactive extract: cytotoxicity, and in vitro wound healing property, fibroblast cell culture	[67,76]
*P. aculeata* leaves	Soxhlet pre-extraction to remove lipids followed by hot water extraction	Ethanol, followed by water	Mucilage: emulsifier	[77]
*P. aculeata* leaves	Simple stirring	Water	Bioactive extract: microencapsulation and Caco-2 cell line viability	[68]
*P. aculeata* leaves	Maceration followed by fractionation in solvents by polarity order	Methanol (maceration), hexane, dichloromethane, ethyl acetate, and butanol (fractions)	Bioactive extract: cytotoxicity against human promyelocytic leukemia (HL60) and human breast adeno-carcinoma (MCF-7) cells	[8]
*P. aculeata* leaves	Supercritical fluid extraction, pressurized liquid extraction, and Soxhlet	CO_2_, ethanol, water, hexane	Bioactive extract: acetylcholinesterase (AChE) and lipoxygenase (LOX) inhibitory activities	[36]
*P. aculeata* leaves	Simple stirring	Water	Mucilage: pigment clarification with activated carbon	[78]
*P. aculeata* leaves	Maceration and fractionation	Methanol (maceration), hexane (fraction)	Bioactive extract: topical anti-inflammatory activity on acute and chronic inflammation	[10]
*P. aculeata* leaves	Hot homogenization	Water	Mucilage: emulsifier	[79]
*P. aculeata* leaves	Simple stirring	Water	Mucilage: fat replacer in processed mortadella meat	[80]
*P. aculeata* leaves	Hot homogenization	Water	Mucilage: material for biodegradable films	[81]
*P. aculeata* leaves	Hot homogenization	Water (extraction) and ethanol (fiber remotion)	Mucilage: emulsifier	[82]
*P. aculeata* leaves	Hot homogenization	Water	Mucilage: emulsifier and encapsulating agent for α-tocopherol	[83]
*P. aculeata* leaves	Simple stirring	Water and ethanol	Bioactive extract: antibacterial activity (*Enterococcus faecalis*, *Streptococcus mutans*, and *Lactobacillus casei*)	[84]
*P. grandifolia* leaves	Microwave-assisted extraction and Soxhlet	Water and ethanol	Bioactive extract: FRAP, ABTS, DPPH, and total phenolic content	[85]
*P. grandifolia* leaves	Simple stirring	Methanol	Bioactive extract: acute oral toxicity in mice	[86]
*P. grandifolia* leaves	Maceration and fractionation	Methanol (maceration), hexane, ethyl acetate, and water (fractions)	Bioactive extract: β-Carotene bleaching, FRAP, DPPH, total phenolic content	[87]
*P. grandifolia* leaves	Simple stirring	Methanol	Bioactive extract: cytotoxicity against nasopharyngeal epidermoid carcinoma (KB), cervical carcinoma (CasKi), colon carcinoma (HCT 116), hormone-dependent breast carcinoma (MCF7), lung carcinoma (A549), and the non-cancer human fibroblast (MRC-5) cell lines	[46]
*P. grandifolia* leaves	Infusion and maceration	Water (infusion) and ethanol (maceration)	Bioactive extract: acute and prolonged diuretic activity in Wistar rats	[88]
*P. grandifolia* leaves	Simple stirring, reflux, and maceration	Ethanol and water	Bioactive extract: antioxidant activity (DPPH, FRAP) and antibacterial activity (*Staphylococcus aureus* and *Pseudomonas aeruginosa*)	[69]
*P. grandifolia* leaves	Maceration	Ethanol and water, hexane, dichloromethane, chloroform, ethyl acetate, and methanol	Bioactive extract: antioxidant activity (DPPH, FRAP) and antibacterial activity (*S. aureus*, *Escherichia coli*, *Bacillus subtilis*, and *Pseudomonas aeruginosa*)	[89]

## 7. Mucilage Characteristics and Composition

**Figure 3:**
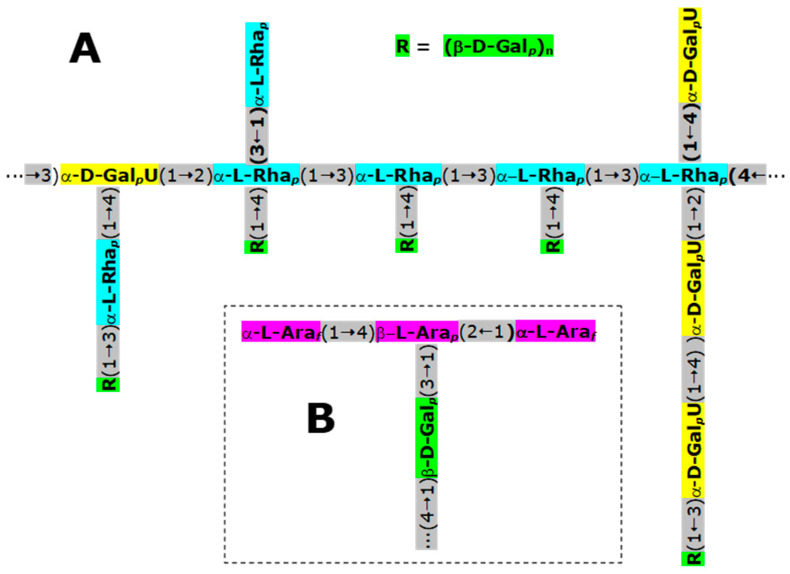
Some structural features described for the polysaccharide found in the mucilage of *Pereskia aculeata* leaves [93,94]. Ara = arabinose; Gal = galactose; GalU = galactouronic acid; Rha = rhamnose; the subscripts *p* and *f* denote the pyranosyl and furanosyl forms, respectively.

Plant mucilage is a renewable and cost-effective source of plant-based compounds that are biologically active, biodegradable, biocompatible, nontoxic, and environmentally friendly. They have a wide range of applications in food, nutraceuticals, and cosmetics [90]. Mucilage is a gelatinous type of hydrocolloid that interacts very strongly with water [91]. It is composed mainly of polysaccharides, but also contains glycoproteins, tannins, alkaloids, and steroids [92]. Upon hydrolysis, mucilage produces an indefinite number of monosaccharides, such as L-arabinose, D-xylose, L-rhamnose, and galacturonic acid, which in the original structure are linked in various ways. Structural studies have been done with the mucilage from *P. aculeata* leaves [93,94]. A water-soluble heteropolysaccharide was identified that contains 3.5% protein. The polysaccharide is composed of arabinose, galactose, rhamnose, and galacturonic acid in a molar ratio of 5.1:8;2:1.8:1.0. It also contains O-acetyl groups in its surface [95], a feature that affects its behavior in solution. Figure 3 shows the structure of two fragments obtained by partial acid hydrolysis. Panel B reveals that the polysaccharide has a (1 → 4)-linked β-galactopyranosyl chain partially substituted at O-3 by β-L-arabinopyranosyl units. The latter are in turn substituted by non-reducing groups of α-L-arabinofuranosyl units. A striking feature is also provided by the O-substituted units of galacto-pyranosyluronic acid (panel A in Figure 3), which are linked (1 → 2) to the rhamno-pyranosyl units. Proteins of the *P. aculeata* leaves’ mucilage can be linked covalently to the polysaccharide. This involves arabinose units that are connected to hydroxyproline residues in the protein [94].

A method of using water at room temperature to obtain ora-pro-nobis mucilage has been proposed [77]. The procedure consists of the following steps: (1) immersion in 99% ethanol for 72 h (lipids and pigments remotion); (2) drying of the leaves (25 °C); (3) hot extraction in Soxhlet with ethanol; (4) drying of the leaves; (5) aqueous extraction followed by centrifugation (insoluble fraction removal); (6) precipitation of polysaccharides with ethanol and drying; (7) polysaccharide re-dispersion in water (25 °C for 16 h); (8) centrifugation; (9) treatment of the supernatant with a NaCl solution (to prevent co-precipitation of proteins) and ethanol addition; (10) centrifugation; (11) washing of the precipitate with ethanol and drying at 25 °C to obtain the mucilage. Analysis of the polysaccharides by GC-MS and NMR techniques revealed the presence of galactose, arabinose, rhamnose, fucose, and partially esterified galacturonic acid, whose main chain consisted of β-D-galactose in 1 → 4 linkage, partially substituted at C-3 by arabinose. This characterizes a type I arabinogalactan, though fucose units are equally present.

The yield of mucilage extraction from ora-pro-nobis is generally in the range of a few percent. The yield of a mucilage extraction from *P. aculeata* leaves, for example, was reported to be equal to 1.6% (wt/wt) with a zeta potential of −25 mV (at pH 5.5) in water [77]. The authors attributed the surface charge to the presence of carboxyl groups in the structure of galacturonic acid and to amino acid side chains. They also observed a decrease in surface tension with increasing mucilage concentrations and related this behavior to the presence of *O*-acetyl and *O*-methyl groups in the polysaccharides and to amino acid side groups of the proteins. This mucilage can act as a hydrocolloid surfactant, since these groups can induce polarity changes in the surface (less polar).

The purification of ora-pro-nobis mucilage was also accomplished using a preparative chromatographic technique with a polyacrylamide cryogel synthesized with a L-tryptophan or L-phenylalanine surface immobilization [83]. The authors reached values of up to 94.45% of protein recovery from the mucilage after this one-step purification procedure.

The mucilage from the green fruit of *P. aculeata* was purified with a yield of approximately 1% (10.0 ± 0.5 g mucilage/kg green fruit) [71]. This is somewhat less than the yield obtained with *P. aculeata* leaves (1.6%) [77]. However, it is worth mentioning that the mucilage powder extracted from the fruit contains approximately twice the protein content than the mucilage extracted from the leaves [71].

The rheological properties of hydrocolloids can be considered one of their most important functional characteristics. In this respect, the effects of sodium chloride (NaCl), calcium chloride (CaCl_2_), and sucrose on the rheological properties of *P. aculeata* (leaves) mucilage were investigated [96]. The investigation detected a Newtonian behavior for the sample prepared with 1.25% mucilage. On the other hand, samples with 2.5%, 3.75%, and 5% mucilage showed a non-Newtonian behavior, fitting to the power law rheological model, with pseudoplastic behavior. A pseudoplastic fluid is a fluid that increases viscosity as force is applied. The authors also observed that the addition of NaCl or sucrose did not affect the shear thinning behavior of ora-pro-nobis mucilage, but the presence of these additives impacted the viscosities of the systems. Other investigations on the mucilage extracted from the leaves led researchers to classify it as pseudoplastic [79,97]. Increasing mucilage concentration had a positive effect on viscosity, while increasing temperature had a negative effect.

The emulsification capacity of the ora-pro-nobis mucilage was the object of several investigations. One such investigation tested the ora-pro-nobis mucilage for its application as an emulsifying agent in emulsified and cooked meat products (mortadella-type meat product) [80]. The capacity of emulsion formation and its stability when subjected to different temperatures (25 °C and 80 °C meat product cooking temperature) were evaluated. There was no significant difference between room temperature and 80 °C treatments for emulsion stability, thus revealing an important result for the maintenance of emulsion stability during the processing steps.

Nano-emulsions using ora-pro-nobis mucilage as an emulsifier have been prepared by ultrasonication [82]. It was inferred that to reach Sauter mean diameters (d_32_) smaller than 200 nm, the mucilage concentrations should be smaller than 2%, along with a high-power amplitude (70–90%) and shorter emulsion sonication time. Also, the treatment containing 1.5% ora-pro-nobis mucilage and 1.0% soybean oil conferred improved kinetic stability considering that low mean droplet diameter values persisted during the 30 days of storage time.

The formulation of biodegradable films also has been investigated using ora-pro-nobis mucilage [98]. In a starch-based film with 30% of ora-pro-nobis mucilage added, compact microstructures were identified without cracks and with small, suspended particles. It was concluded that this behavior may indicate a strong interfacial adhesion between starch and mucilage at this concentration. Mucilage concentrations of 10 and 20% resulted in rough surfaces and the formation of agglomerates, which could be caused by incomplete solubilization of the polymers. Rheological analysis revealed an increase in solution viscosity when the mucilage was incorporated into the 30% concentration. The interfacial adhesion between starch and the mucilage molecules can be explained by the latter presenting a macromolecular structure with long chains. Thus, the highest concentration that was evaluated (30% of mucilage) provided greater inter-molecular interactions and changes in chain entanglement. This could be a positive characteristic since the development of specific interactions decreases the macromolecular mobility necessary for crystallite formation, which can help to prevent starch retro-gradation.

## 8. Small Molecular Weight Bioactives of *P. aculeata* e *P. grandifolia:* Extraction Methods and Properties

**Figure 4:**
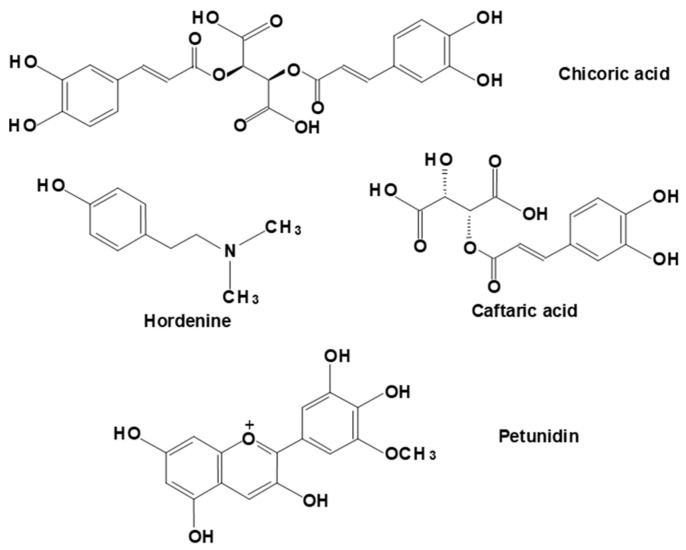
Representative structures of polyphenolic compounds found in ora-pro-nobis in addition to the phenylethylamine alkaloid hordenine.

Phenolic compounds, alkaloids, carotenoids, triterpenoids, and steroids are some of the small molecules found in *Pereskia* sp. extracts and are presented in Table 5. Ora-pro-nobis leaves contain several bioactive compounds such as hordenine, di-tert-butylphenol, petunidin, quercetin, cis-caffeic acid, trans-caffeic acid, caffeic acid, rutin, isorhamnetin, and some glycosylated derivatives of these compounds [6,11]. The structures of a few of these compounds, selected because they seem not to be of universal occurrence among plants, are shown in Figure 4. The unusual phenylethylamine alkaloid hordenine has been recently associated with several bioactive and pharmacological properties including anti-inflammatory, anti-diabetic, and anti-fibrotic effects [11].

An ethanolic extract of *P. aculeata* was evaluated regarding its cytotoxicity in lettuce seeds at different concentrations [48]. The lettuce model was chosen because it is considered a sensitive species and a widely used plant in toxicity research. Plant cytogenetic bioassays are an important tool to evaluate possible toxic effects because they frequently correlate fairly well with mammalian test systems and also because they provide evidence of the effects of substances at the chromosome level and on the cell cycle. The results showed that the *P. aculeata* extract did not affect germination and the mitotic index. It affected, however, lettuce root and shoot growth. The opinion was expressed that these results do not affect the potential applicability of ora-pro-nobis.

Cultivation of *P. aculeata* in diverse soil types (sand, crude soil, sand and soil, sand and cattle manure, soil, and cattle manure) was done, aiming at correlating the effects of their nutritional contents with mucilage production and wound-healing capacity [67]. The results showed that the substrate (soil type) used in the cultivation interferes with mucilage production, the highest production having been obtained in the cultivations that were done with the least nutritive substrates (sand and crude soil). However, substrates did not interfere with cytotoxicity and wound healing, both tested with L929 mouse fibroblast cells.

A biorefinery approach for improving the selective extraction of *P. aculeata* leaves was proposed [99]. The process aims at the full use of *P. aculeata* leaves, is based on green compressed fluids, and consists of various types of extraction in sequence. The following extraction sequence was applied to the same leaf material: (1) supercritical fluid extraction (SFE) with CO_2_ or SFE + CO_2_:ethanol; (2) liquid extraction with gas expansion (GXL), using CO_2_:ethanol, or pressurized liquid extraction (PLE) with ethanol (GLX and PLE were compared); and (3) subcritical water extraction (SWE). The following compounds were recovered in each process step: (1) terpenoids, (2) carotenoids and phenolic compounds, (3) and proteins and carbohydrates. The final residue was mainly composed of fibers. The compounds identified by employing liquid chromatography coupled to diode array detection and electrospray ionization mass spectrometry (tentative identification) are presented in Table 5. The total carotenoid contents (in mg lutein equivalents per g extract) were equal to 66.88 ± 0.01 for the CO_2_ SFE extract, 62.05 ± 1.27 for the CO_2_ + ethanol SFE extract, 56.69 ± 0.16 for the GXL extract, and 58.73 ± 1.17 for the PLE extract. It was concluded that the supercritical CO_2_ extraction presents selectivity towards the carotenoids. Conventionally, these compounds are recovered by organic solvents (non-polar solvents), so the results have proven that SFE is an effective method for carotenoid extraction. In another work of the same research group, the inhibitory effect of an extract of *P. aculeata* leaves on acetylcholinesterase, as well as its anti-inflammatory effect, was determined [36]. Among the extracts obtained (SFE with CO_2_ at different temperatures and Soxhlet with hexane), those obtained with Soxhlet (IC_50_ = 197.2 ± 25.3 μg/mL) and SFE at 50 °C (IC_50_ = 174.6 ± 52.8 μg/mL) presented moderate inhibitory potency against acetylcholinesterase. Furthermore, the IC_50_ for anti-inflammatory activity, inferred from the lipoxygenase (LOX) inhibition, was equal to 163.9 ± 6.5 3 μg/mL for the SFE extract obtained at 50 °C.

Sequential extraction of *P. aculeata* leaves with organic solvents with increasing polarity, petroleum ether < chloroform < methanol, was used as a strategy for attributing biological activities to groups of substances with different polarities [9]. It was found that the petroleum ether extract inhibited the growth of all tested strains and exhibited potent antibacterial activity against *Escherichia coli* (2 µg extract/mL). The chloroform extract showed inhibitory activity against *Bacillus cereus* and *Staphylococcus aureus*. In relation to antifungal activity, petroleum ether and methanol extracts were more effective in inhibiting the growth of *Aspergillus versicolor* (2 µg extract/mL). The authors of the aforementioned study did not provide an explanation for the different performances of petroleum ether and methanol extracts. However, the methanol extracts presented higher contents of polyphenolics (30% more) when compared to the petroleum ether extracts. This difference may be at least in part related to the better antimicrobial performance of the former, but there are certainly other differences in composition between both extracts to be considered. Cytotoxicity of the extracts was tested with SH-SY5Y human neuroblastoma cells. The IC_50_ values were >200 µg/mL for all extracts, considered non-cytotoxic when judged by the criteria set by the National Cancer Institute, which states that extracts with IC_50_ < 20 µg/mL can be considered toxic to cells [102]. It is interesting to note that the methanolic extract (100 µg extract/mL) was able to reduce significantly the adenylate cyclase 1 expression (ADCY1) in neuroblastoma cells. ADCY1 is a neurospecific synaptic enzyme that upregulates the cAMP signaling cascade. It is stimulated by Ca^2+^ and plays an essential role in neurodevelopment and neuroplasticity [103]. The overexpression of ADCY1 in the mouse forebrain caused enhanced ERK1/2 (extracellular regulated kinase) activation and reduced sociability [104]. The authors highlighted that clinical and epidemiologic research suggests that the reduction of ADCY1 expression has well-documented benefits, including benefits for heart disease and pain [105].

Evaluation of the phenolic profile of *P. acuelata* leaf extract [6] identified caftaric acid as the main phenolic constituent, followed by quercetin-3-*O*-rutinoside and isorhamnetin-*O*-pentoside-*O*-rutinoside. Furthermore, it is worth mentioning that the extract showed higher antioxidant activity than Trolox in the DPPH and ABTS assays in addition to antibiotic activity. The authors attributed the bioactivity of the extract to the presence of phenolic compounds and associated the presence of rutin with its antibacterial activity. Caftaric acid (see Figure 4) is an ester form of caffeic acid and has been related to antioxidant and anti-inflammatory activities [106].

Most hitherto-discussed studies have analyzed the ora-pro-nobis leaves, but there are also studies in which the phenolic compounds profile from other parts, such as ripe fruits [99], were analyzed. The fruits examined had relevant amounts of protein, calcium, caffeic acid, rutin, hesperidin, 2,4-dihydroxybenzoic acid, 2,5-dihydroxybenzoic acid, p-coumaric acid, quercetin, and isoquercitrin, and were low in fat. Seeds were also analyzed in the same study and shown to contain galangin and ferulic acid. Green ora-pro-nobis fruits also contain high protein levels and mucilage [71]. High digestibility (75% to 80%) has been attributed to the ora-pro-nobis proteins [107]. As they have high protein and low fat content, it can be said that ora-pro-nobis fruits have a high capacity for food integration.

**Figure 5:**
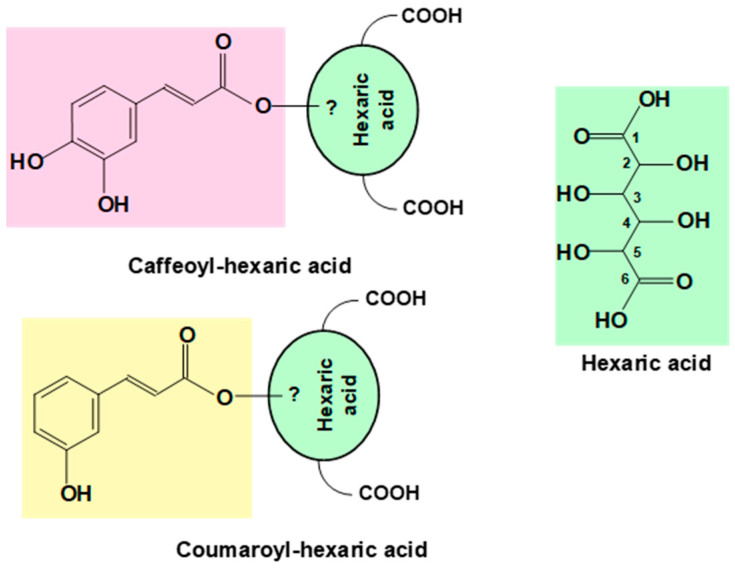
Structural features of caffeoyl hexaric acid and coumaroyl hexaric acid reported to occur in a hydroalcoholic extract from *P. aculeata*, highlighting the uncertainty about the hydroxyls of hexaric acid involved in the formation of the ester linkage [73].

The presence of isorhamnetin, chicory acid, two isomers of caffeoyl hexaric acid, and two isomers of coumaroylhexaric acid in a hydroalcoholic extract from *P. aculeata* was reported [73]. The exact structure of these compounds still remains undefined, as the carbons in the hexaric acid moiety that are involved in the ester bonding were not yet established, a fact that is illustrated by Figure 5. In relation to the extract activity, the inhibition of lipid peroxidation was considered moderately efficient (up to 32% inhibition), together with hypotonic hemolysis inhibition and H_2_O_2-_induced hemolysis inhibition. Effects of the extract on cell viability and levels of intracellular ROS were evaluated in cancer (A549, Caco-2, HepG2) and normal (IMR90) cell lines. The extract exhibited no in vitro toxicity against both cancer and normal cell lines (within the concentration range tested). It is recognized that moderate oxidative stress contributes to neoplastic transformation, both by decreasing genome stability and by providing ROS signaling that stimulates cell proliferation [108]. It was shown experimentally [73] that the extract displayed intracellular antioxidant activity in both malignant (A549, HepG2, and Caco-2) and healthy (IMR90) cells, since it decreased ROS generation. This action was correlated with the isorhamnetin extract’s content, one of the main phenolic compounds that were quantified (1060 mg/100 g), and with rutin (1078 mg/100 g), which was found in all samples. This reinforces the notion that the last compound has a wide range of pharmacological uses (such as antimicrobial, antifungal, and antiallergic uses) mainly due to its substantive antioxidant properties, especially as a free radical scavenging agent [109].

The topical anti-inflammatory activity of the hexane fraction obtained from a methanolic extract of *P. aculeata* leaves was evaluated in models of acute and chronic ear dermatitis in mice [10]. Mice ear edema was induced by topical application of croton oil, arachidonic acid, capsaicin, ethyl-phenylpropiolate, and phenol, and by a subcutaneous injection of histamine. Histamine is an endogenous vasoactive amine that is released by mast cell degranulation and increases vasodilation and vascular permeability during the inflammatory process, leading to plasma leakage, erythema, and nerve fiber sensitization. Furthermore, histamine regulates the release of several cytokines, IL-1β, TNF-α, and IL-6 [110]. Histopathological analysis was also performed to evaluate the extract activity in a croton oil multiple application test. In addition, an acute dermal irritation/corrosion test in rats was accomplished. Results showed that the extract and dexamethasone (positive control) showed similar activities in the croton oil-induced ear edema multiple application test. The histopathological analysis emphasized that the ora-pro-nobis extract inhibited the edema considerably. Furthermore, leukocyte infiltration and vasodilation were also less intense in the extract-treated group. Finally, the animals subjected to topical application of ora-pro-nobis extract in the acute dermal irritation/corrosion test showed no clinical signs of local or systemic toxicity.

The main carotenoids found in ora-pro-nobis extracts are listed in Table 5. The results indicate that there is an increase in carotenoid concentration after ora-pro-nobis leaves are submitted to thermal treatments, such as cooking, microwaving, or steaming [38,98]. Furthermore, cooking increases the lutein and the α- and β-carotene levels in leaves [98]. The steam treatment for 4 min promoted the greatest increase in the levels of these carotenoids and in the level of vitamin A in food (788.1 µg/100 g). This is allegedly [98] in line with several studies that demonstrate the influence of home cooking on carotenoid content, as the physical state and location of carotenoids in plant materials affect the release of these compounds, making them available. On the other hand, stir-frying led to a higher degradation of total carotenoids relative to the raw sample (49%) [39].

**Figure 6:**
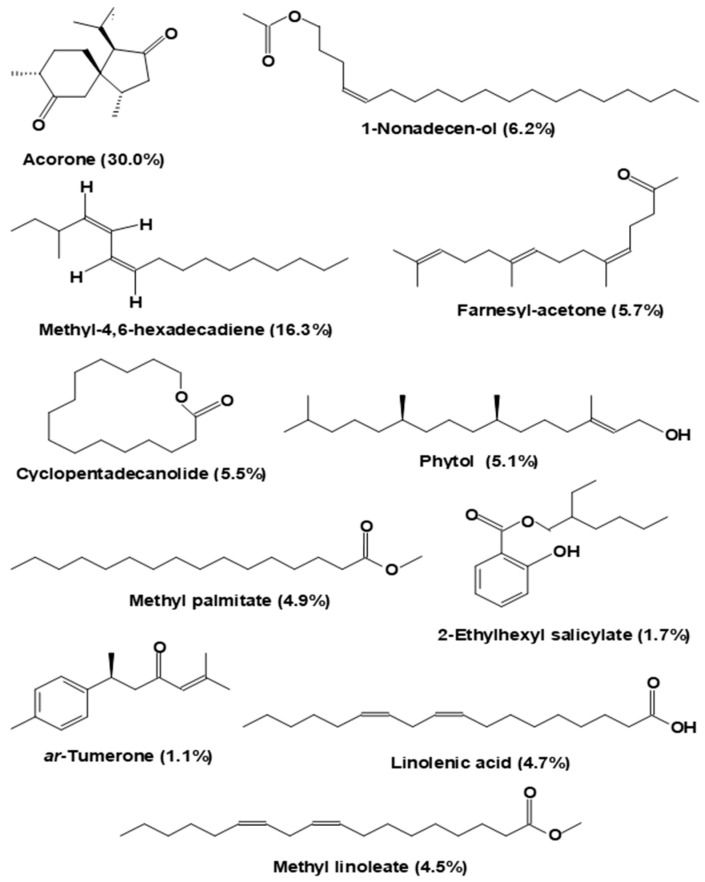
Structures of the most abundant molecular components of the essential oil fraction of the aerial parts of *P. aculeata* [9].

Characterization of the essential oils in the ora-pro-nobis species is still at its beginnings. Figure 6 shows the structures of the most abundant essential oils that were isolated by hydro-distillation of the aerial part of *P. aculeata* [9]. Regarded as a whole, the compounds present considerable structural variability, ranging from oxygenated sesquiterpenes to long-chain fatty acids or derivatives. A total of 24 compounds were identified. Acorone, an oxygenated sesquiterpene, was by far the most abundant (30%). A previous study, however, identified phytol as the most abundant compound in the same species (29.4%) [14], a compound much less abundant among those illustrated in Figure 6 [9]. Essential oil content in ora-pro-nobis leaves seems, thus, to vary greatly in quality and abundance with the environmental conditions, age of the plant, method of harvesting, and even with the method used for isolation.

Studies on the application of ora-pro-nobis essential oils to human health or well-being are very scarce. There has been a recent attempt at preparing a vegan facial cream with ora-pro-nobis essential oil in combination with *Aloe vera* components [111]. The preparation was apparently successful in terms of its physical properties, but no attempts have been reported so far at testing its efficacy in biological terms. An attempt at exploring the antimicrobial properties of the ora-pro-nobis essential oils has also been reported [112]. In this work, the antimicrobial capacity of the essential oils against microorganisms commonly found in the coats of health professionals was investigated. The research revealed bacteriostatic capacities against several strains of *Staphylococcus* and *Bacillus*, Actinobacterium phylum, as well as against a non-fermenting gram-negative bacillus. Resistant *Staphylococcus* strains were also detected, however, suggesting that for bacteriostatic purposes, the use of ora-pro-nobis essential oils can only be used in combination with other active agents.

## 9. Conclusions and Perspectives

*P. aculeata* and *P. grandifolia* are native species of the Atlantic Forest in Brazil. These plants are shrubs or climbers that occur in a variety of soils. Ora-pro-nobis leaves are an important source of nutrients and can be used as an unconventional edible plant, as they have a high concentration of proteins, fiber, and iron, and have a low fat content. Furthermore, they have mucilage on their leaves, and can thus be used as an emollient in folk medicine and consumed as a food source.

The information reported here not only corroborates the importance of the production and consumption of *P. aculeata* leaves, but also reinforces their potential as a sustainable source of nutraceuticals and promising food ingredients to be used for food enrichment.

This review presented work carried out with two species occurring in Brazil (*P. aculeata* and *P. grandifolia*), pointing out different ways of extracting flour from leaves, mucilage, and ripe and green fruit. Furthermore, its bioactivity was exposed through the characterization of its phenolic compounds and their antioxidant, anticarcinogenic, anti-inflammatory, and antimicrobial activity. All these observations highlight its great importance as a nutraceutical agent.

As seen in this literature review, most of the studies evaluated ora-pro-nobis leaves, mucilage, and flour. In the works in which these extracts were applied to foods, only the approximate composition and sensory acceptance were evaluated. This reinforces the need for new studies in which food formulations containing ora-pro-nobis are characterized by other analyses, such as texture determination and color evaluation, for example. Many chemical compounds have been identified, and several of them seem not to be very common in plants. However, frequently, there are crucial uncertainties about their structures, i.e., many studies are still needed to confirm important details about the chemical components of ora-pro-nobis. It is also important to emphasize the need for further studies exploring the fruit and flowers of *P. aculeata*, as they are scarce in the literature. Furthermore, the works that were found used and analyzed predominantly the species *P. aculeata*, with only a few having evaluated *P. grandifolia*. A more equalized use of both species would be highly desirable.

Finally, an important gap is safety and toxicity, topics to which little attention has yet been devoted. Although some limited observations are available with respect to laboratory animals, data on humans are clearly absent. The same can be said about the microbial safety and risk of pathogens as a consequence of fresh consumption. Approaches to these aspects would, thus, be highly desirable in future research.

## Figures and Tables

**Table 5:** Main molecules identified in *P. aculeata* and *P. grandifolia* extracts reported in the literature.

Ora-Pro-Nobis Species	Extraction Solvent	Extraction Technique	Major Compounds Identified	Reference
*P. aculeata* leaves	Ethanol:water (60:40)	Simple stirring	Phenolic compounds: caftaric acid; chicoric acid; caffeoyl-hexaric acid; coumaroyl-hexaric acid; quercetin-pentoside; kaempherol-rhamnoside-hexoside	[73]
*P. aculeata* leaves	Aqueous and ethanolic extracts	Subcritical water extraction and pressurized liquid extraction	Phenolic compounds: caftaric acid isomers; quercetin-*O*-pentoside-*O*-rutinoside; quercetin-3-*O*-rutinoside; isorhamnetin-*O*-pentoside-*O*-rutinoside; quercetin-glucoside; kaempferol-3-*O*-rutinoside; isorhamnetin-3-*O*-rutinoside; isorhamnetin-3-*O*-glucoside	[85,99]
*P. aculeata* leaves	Ethanol:water (70:30)	Simple stirring	Phenolic compounds: caftaric acid; trans caftaric acid; caffeic acid derivative quercetin-*O*-pentoside-*O*-rutinoside; quercetin-*O*-pentoside-*O*-hexoside; quercetin-3-*O*-rutinoside; isorhamnetin-*O*-pentoside-*O*-rutinoside; isorhamnetin-*O*-pentoside-*O*-hexoside; kaempferol-3-*O*-rutinoside; isorhamnetin-3-*O*-rutinoside	[6]
*P. grandifolia* leaves	Extracted with methanol and fractionated with hexane		Decane; 4,5-dimethyl-2,6-octadiene; undecane; nerolidol; 1-methoxy-p-tolyl-propan-2-ol; neophytadiene; methyl palmitate; palmitic acid; linolenic acid methyl ester; phytol; methyl octadecanoate; tricosane; cholesterol; campesterol; stigmasterol; sitosterol; taraxasterol taraxerol;	[11]
*P. grandifolia* leaves	Ethanol, water, and hexane	Microwave-assisted extraction and Soxhlet extraction	Phenolic compounds: 4-aminobenzoic acid; 4-hydroxymethylbenzoic acid; caffeic acid; chlorogenic acid; cinnamic acid; ellagic acid; ferulic acid; gallic acid; mandelic acid; p-anisic acid; p-coumaric acid; protocatechuic acid; salicylic acid; sinapic acid; syringic acid; vanillic acid; epicatechin; kaempferol; myricetin; quercetin; rutin; syringaldehyde; vanillin, umbelliferone	[36]
*P. aculeata* leaves	Acetone	Maceration	Carotenoids: 9-cis-neoxanthin; 13-cis-violaxanthin; all-trans-neochrome; 13-cis-lutein; 15-cis-lutein; all-trans-lutein; all-trans-zeaxanthin; all-trans-β-cryptoxanthin; 13-cis-β-carotene; all-trans-α-carotene; di-cis-β-carotene; 9-cis-β-carotene; all-trans-β-carotene	[39]
*P. grandifolia* leaves	Water	Cooking in boiling water, water vapor and microwave	Phenolic compounds: caffeic acid; chlorogenic acid; catechin; rutin; *p*-coumaric acid; *t*-ferulic acid; luteolincarotenoids: lutein, α-carotene, β-carotene	[100]
*P. aculeata* *Fruit*			2,4-DHBA, 2,5-DHBA, caffeic acid, chlorogenic acid, ferulic acid, catechin, hesperidin, isoquercitrin, *p*-coumaric acid, quercetin, and rutin.	[101]

## Data Availability

The datasets supporting the conclusions of this article are included within the article.
